# Regarding: ‘Epoetin *β* treatment in patients with cancer chemotherapy induced anaemia: the impact of initial haemoglobin and target haemoglobin levels on survival, tumour progression and thromboembolic events’

**DOI:** 10.1038/sj.bjc.6605474

**Published:** 2009-12-08

**Authors:** G Schwarzer

**Affiliations:** 1 Institute of Medical Biometry and Medical Informatics, University Medical Center, Freiburg, Germany

In the present study (Aapro *et al*, 2009), results of an updated meta-analysis of 12 randomised controlled trials with 2297 patients are presented on the effects of Epoetin *β* on survival, tumour progression and thromboembolic events (TEEs) in cancer patients. Special emphasis is given to the impact of different haemoglobin (Hb) levels at initiation of Epoetin *β* treatment. Overall, the risk for death was increased by a factor of 1.13 (95% CI 0.87–1.46) and that for thromboembolic events was increased by a factor of 1.62 (95% CI 1.13–2.31) in patients receiving Epoetin *β* compared with controls. When the analysis was limited to patients receiving Epoetin *β* at Hb⩽10 g dl^−1^, the authors identified no evidence for a negative impact on survival (Epoetin *β* *vs* control HR 0.99, 95% CI 0.70–1.40) and a favourable effect for disease progression (HR 0.73, 95% CI 0.57–0.94). However, the authors describe an increased risk for death in patients with Hb levels of > 11 g dl^−1^ at initiation of Epoetin *β* therapy.

To evaluate the effect of baseline haemoglobin level on overall survival and other outcomes, Aapro *et al*. stratified the data by entry Hb and compared the treatment estimates in such created subgroups. Based on this analysis, the authors conclude that no detrimental effect of Epoetin *β* on survival or tumour progression is apparent when initiated at Hb levels up to 11 g dl^−1^. However, no statistical test for heterogeneity between subgroups was conducted. Accordingly, it is debatable whether the observed differences in treatment effects between entry Hb subgroups is due to entry Hb acting as an effect modifier or is just a play of chance. For two subgroups, a *Z* test on heterogeneity (Borenstein *et al*, 2009) can be calculated from estimated treatment effects and width of confidence intervals in the respective subgroups. The *Z* test on heterogeneity is based on the difference in the two estimated treatment effects divided by a pooled standard error. Information to conduct this test is given in Figure 2 of Aapro *et al*. for overall survival and other outcomes. For overall survival, a *Z* test for heterogeneity comparing the subgroups entry Hb⩽11 g dl^−1^ and entry Hb>11 g dl^−1^ would yield a *P*-value of 0.65. This test would also be non-significant for progression-free survival (*P*=0.36) and time to TEE (*P*=0.90). Bohlius *et al* (2009) also evaluated baseline Hb level as a potential effect modifier of treatment with erythropoiesis-stimulating agents in a larger dataset with 13 407 cancer patients using a Cox regression model stratified by study. The analysis with five Hb subgroups yielded a *P*-value of 0.75.

The authors consider target haemoglobin level as a potential effect modifier. Target Hb level was defined as the maximum Hb level actually achieved up to 28 days after the end of treatment, which is time-dependent information. Using the maximum Hb level during follow-up for stratification is problematic for several reasons. Patients are divided into subgroups based on future information, which requires a specialised methodology in survival data with censored observations. Furthermore, the maximum Hb level during follow-up is dependent on the Epoetin *β* dosage, as well as other factors such as disease severity and number of blood transfusions. Accordingly, as correctly pointed out by the authors in the discussion, results of the analysis of target Hb level need to be interpreted with caution because of methodological limitations and potential confounding (see also Altman and de Stavola, 1994). In addition, interpretation of the results for maximum Hb level achieved is difficult, as it is unclear which effect is actually estimated in subgroups that are created by stratifying on information that is not available before the follow-up ends, either because the patient may die or is lost to follow-up. A more interpretable analysis of target Hb levels could be based on a Cox regression model containing both baseline Hb level and time-dependent Hb level as covariates (Parmar and Machin, 1995). Furthermore, in such Cox regression models, time-dependent information is modelled appropriately and the effect of future Hb values in addition to the baseline Hb level can be quantified and tested against the null hypothesis that future Hb values do not contain any additional information.

Evaluating the impact of haemoglobin levels on survival and other outcomes in cancer patients treated with Epoetin *β* is an important research topic. The authors give some indications on the safety of Epoetin *β* when used within the Hb intervention and target levels as recommended in the revised European label. However, in order to get a more definite answer on the impact of initial and target haemoglobin levels on survival outcomes, analysis in a larger dataset using appropriate statistical methods for time-dependent information would be essential.
